# Extended Aortic Repair for Acute Type A Aortic Dissection with Rupture and Malperfusion Complicated with Ehlers-Danlos Syndrome

**DOI:** 10.3400/avd.cr.22-00024

**Published:** 2022-06-25

**Authors:** Koki Maekawa, Toshiki Fujiyoshi, Masaki Kano, Yu Nakano, Ryumon Matsumoto, Hitoshi Ogino

**Affiliations:** 1 Department of Cardiovascular Surgery, Tokyo Medical University, Tokyo, Japan

**Keywords:** vascular Ehlers-Danlos syndrome, aortic dissection with rupture, valve-sparing root replacement

## Abstract

The patient was a 54-year-old gentleman with sudden chest pain. He suffered from cardiac tamponade and malperfusion of the left carotid artery and the right lower extremity due to acute type A aortic dissection. Rupture of the aortic root and a huge entry from the transverse arch to the proximal descending aorta were found. Extended repairs of valve-sparing root replacement and total arch replacement with frozen elephant trunk were successfully performed. He was discharged without any complications. He was finally diagnosed as having vascular Ehlers-Danlos syndrome by a genetic examination.

## Introduction

Ehlers-Danlos syndrome (EDS) is a disorder in the metabolism of fibrillary collagen, characterized by easy bruising, thin skin with visible veins, and facial features of thin nose, thin upper lip, small earlobes and prominent eyes. EDS is estimated to occur in 1 in 10,000–25,000 people, of which the vascular type (type IV) of Ehlers-Danlos syndrome (vEDS) accounts for about 5%–10% of cases. vEDS is an inherited disorder of connective tissue, resulting from mutations in the COL3A1 gene coding for type III procollagen. Typical cases of vEDS show characteristic physical findings and tissue weaknesses, such as arterial rupture and aortic dissection.^1,2)^ In general, a tear-oriented surgery has been recommended for acute type A aortic dissection (AAAD). However, with increased experiences of total arch replacement (TAR), its prevalence has recently increased, particularly in conjunction with frozen elephant trunk (FET).^3)^ In the proximal site, in cases with rupture or dilatation of the root or with primary entry in the root, concomitant aortic root repairs are required. Of them, valve-sparing root replacement (VSRR) is much more challenging because of prolonged cardiac arrest and cardiopulmonary bypass (CPB). We report on successful extended repairs of VSRR and TAR with FET for massive AAAD with root rupture and multiple malperfusion in a difficult patient with vEDS.

## Case Report

**Figure 1:**
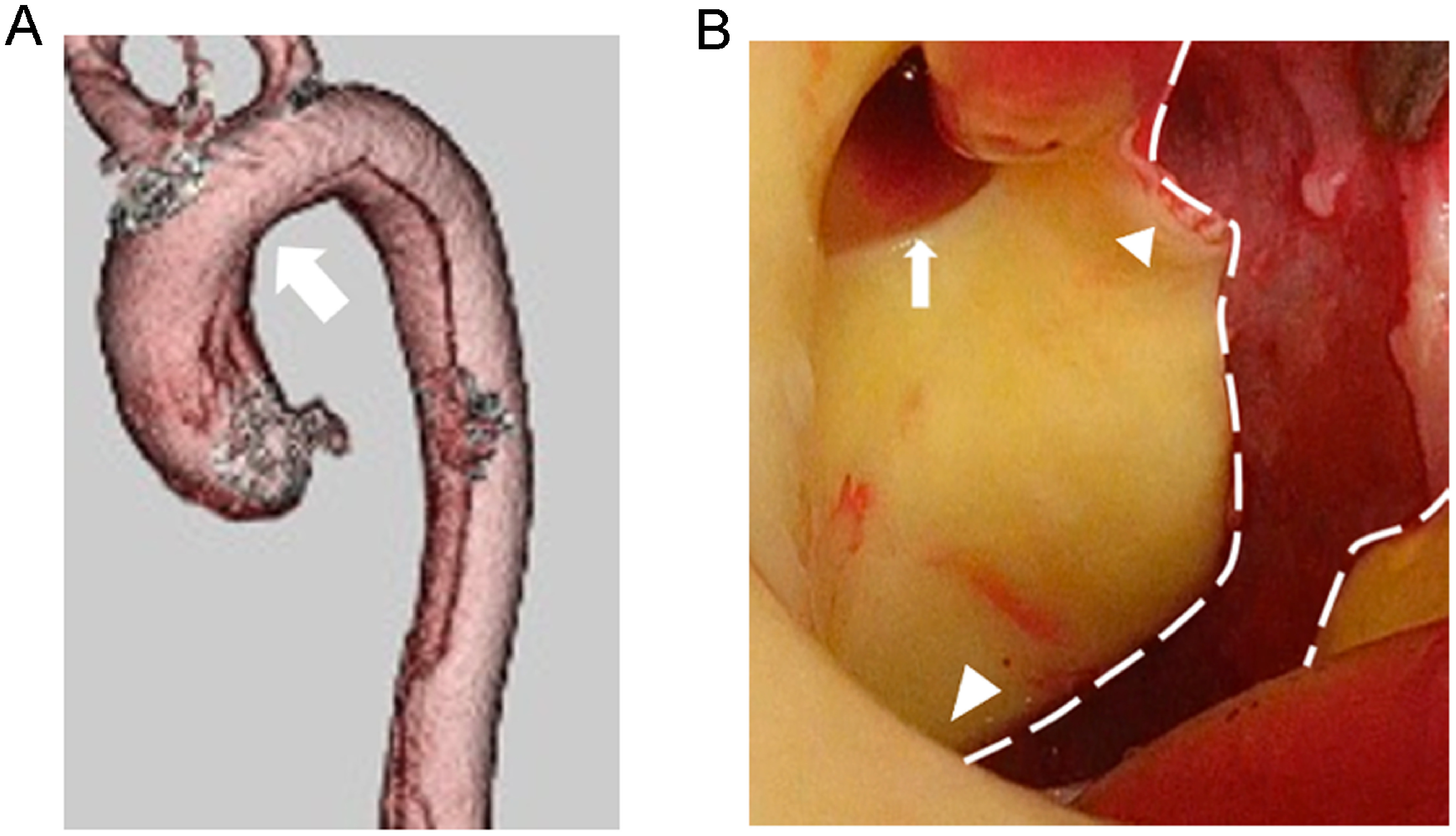
Fig. 1 (**A**) Preoperative computed tomography scan shows a large entry at the transverse arch (arrow). (**B**) A large entry was located from the arch to the proximal descending aorta (a dotted line). The small arrow shows the orifice of the left subclavian artery. The large triangle shows the distal side and the small triangle shows the proximal side of the aorta.

**Figure 2:**
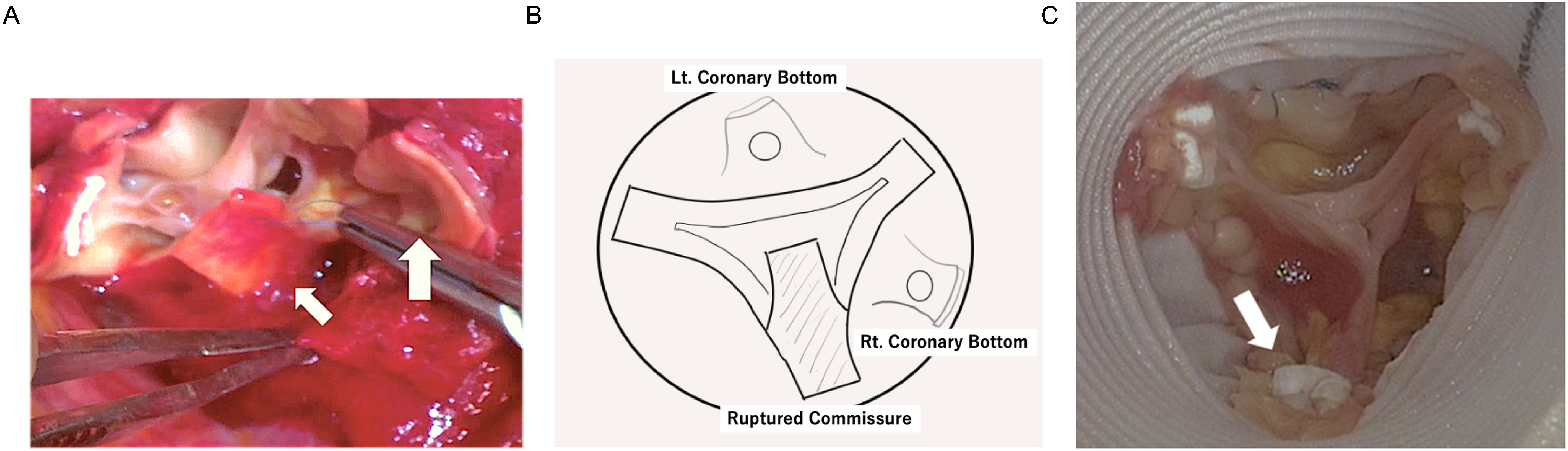
Fig. 2 (**A**) The aortic root was massively dissected with ruptured adventitia around the commissure (small arrow) between the right and noncoronary Valsalva sinuses. The dissection was extended into the right coronary artery (big arrow). (**B**) A scheme of the operative finding. (**C**) Good coaptation of the aortic valve was confirmed with appropriate attachment of the ruptured commissure (arrow) to the graft.

**Figure 3:**
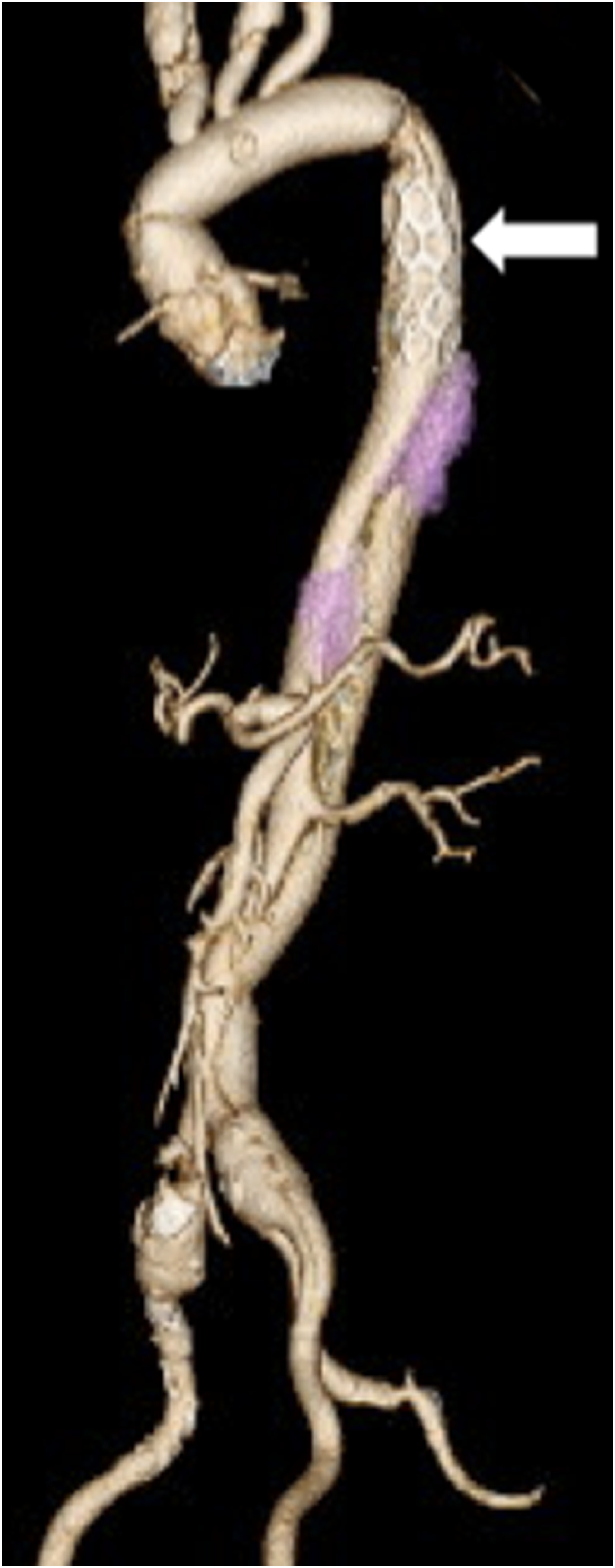
Fig. 3 Postoperative computed tomography scan shows valve-sparing root replacement and total arch replacement using frozen elephant trunk, with closure of the false lumen in the proximal descending aorta (arrow).

The patient was a 54-year-old gentleman. On arrival, his conditions were critical, with a blood pressure of 67/47 mmHg. Echographic examinations showed pericardial effusion and dissecting flaps in the bilateral carotid arteries. Computed tomographic (CT) scans showed AAAD with malperfusion of the left carotid artery and the right common iliac artery. At surgery, CPB was immediately established with a right femoro-femoral circuit. A large amount of clotting was found in the intrapericardial space. Central cannulation for CPB into the true channel of the ascending aorta was added. At 28°C of the bladder temperature, hypothermic circulatory arrest (HCA) was induced and the heart was arrested with retrograde cardioplegia through the coronary sinus. The dissecting ascending aorta was opened. As the CT scans showed, a huge primary entry was located from the transverse arch to the proximal descending aorta at the zone 4 level (**Figs. 1A** and **1B**). The dissection was extended into each arch vessel. The neck vessels were cannulated for antegrade selective cerebral perfusion (SCP), which was maintained at a flow rate of 600–700 ml/min, a blood pressure of 40–60 mmHg, and a perfused blood temperature of 23°C. The proximal descending aorta was transected completely at the zone 4 level, excluding the primary entry. A FET device, FROZENIX (Japan Lifeline Co., Tokyo, Japan), 23 mm in diameter and 60 mm in length, was inserted into the divided descending aorta and its proximal end was fixed roughly to the descending aorta with outside felt reinforcement. To this aortic stump, a multibranched arch graft, Gelweave (TERUMO VASCUTEK, Glasgow, UK), 24 mm in diameter, was connected. Systemic CPB perfusion was commenced through the branch-graft. After systemic reperfusion, the three arch vessels were reconstructed, repairing all dissected arteries. At the proximal site, rupture of the aortic root was found at the commissure between the right and noncoronary Valsalva sinuses (**Figs. 2A** and **2B**). The dissection was extended into the right coronary artery. The aortic valve had no significant deformities, which allowed us to perform VSRR using a reimplantation technique with Gelweave Valsalva Ante-Flo (TERUMO VASCUTEK, Glasgow, UK), 26 mm in diameter. The commissure post between the right and noncoronary cusps had no adventitia. Only the dissecting flap was carefully fixed to the graft with a 5-0 polypropylene pledgeted mattress suture. We added 2nd-low sutures with 5-0 polypropylene mattress suture for attachment of the aortic wall were also meticulously placed (**Fig. 2C**). The coronary buttons were reimplanted to the graft. Particularly, the right coronary artery with the orifice dissected was reattached. The other arch vessels were reconstructed. Finally, the root graft was anastomosed to the arch graft. After de-airing, the aortic clamp was released. The patient was weaned from the CPB. The time of HCA, SCP, myocardial ischemia, CPB, and operation was 80 min, 290 min, 322 min, 415 min, and 15 h, 48 min, respectively. In the postoperative course, he developed delay of full awakening. The CT scans and magnetic resonance imaging revealed a small cerebral infarction on the left frontal lobe and the cerebellum; however, it was not critical and did not cause any permanent neurological dysfunctions. The patient was extubated on the second operative day and he had a normal recovery in the intensive care unit in 5 days. The postoperative CT scans (**Fig. 3**) showed no remarkably abnormal findings and echocardiography showed trivial aortic valve regurgitation. He was finally diagnosed as having vEDS by a genetic test, revealing a novel frame-shift mutation of the COL3A1 gene, and discharged without any symptoms on the 25th operative day. Two years have passed without worsening of AR and the CT scan showed no abnormalities.

## Discussion

vEDS is a rare hereditary syndrome caused by the impaired metabolism of fibrous collagen. Given the vulnerability of the tissue, 80% of patients with vEDS would have some vascular complications by the age of 40 years, and their median life span was reportedly 48 years. Most patients died from aortic dissection or rupture. Although there have been several reports of difficult vascular surgeries, their outcomes were poor because of the vascular frangibility.^1,4,5)^ Recently, the application of FET has been increasing in aortic arch repairs. In particular, for acute aortic dissection, it would enable the dilatation of the true channel with closure of the false lumen and reinforcement of the aortic stump from the inside.^3)^ In this case, TAR with FET was employed, because of critical and extensive aortic dissection with a large entry in the transverse arch to the proximal descending aorta and malperfusion of the right lower extremities. In the presented case, the distal anastomosis of TAR was carried out at zone 4 and the distance between the distal anastomosis (that is, the proximal FET landing) and the left subclavian artery branch was 5 cm. In these settings, given the FET device used, it was quite easy to regulate its distance. However, one of the most serious complications of the FET procedure is spinal cord injury (SCI). In this case, to avoid it, the shortest device of 6 cm length was employed, because the proximal FET landing was at zone 4. Fortunately, the patient did not develop any SCI. The distal end of the FET device was located above the T8 level on the postoperative CT scans. In addition, the enlargement of the downstream true channel was confirmed with the improvement of the lower right limb malperfusion. The use of FET for aortic dissection in Marfan syndrome and other connective tissue diseases is controversial.^6)^ However, good results have been reported,^7)^ and we believe that FET was an effective treatment even if vEDS was diagnosed preoperatively in this case. VSRR was challenging because it is more technically demanding.^8)^ Particularly for vEDS, there are only a few reports about emergency cases with AAAD.^9)^ VSRR was, however, attempted because of the patient’s relatively young age, no significant deformities of the aortic valve, and the surgeon’s (HO) experience in over 200 VSRRs. No adventitia was preserved at the commissure between the right and noncoronary cusps. Careful attachment of such a destroyed and fragile commissure to the graft was required. Apart from remodeling procedures, the commissure posts are inserted in the graft and attached to its inside, which is one of the advantages of reimplantation techniques. During surgery, it was suspected that the patient had a sort of connective tissue disease because of his tissue fragility. Finally, it was revealed as vEDS. This is the first report on a patient with vEDS, with VSRR and TAR with FET for AAAD being successfully performed. Long-term results are important in VSRR for connective tissue diseases including vEDS, and the surgical selection is controversial.^10)^ In this case, the patient had a good 2-year postoperative course. Intensive follow-up is necessary because of the fragility of the tissue.

## Conclusion

vEDS causes various vascular complications because of tissue fragility. In this case, emergency extensive repairs of VSRR and TAR with FET for critical AAAD with rupture and malperfusion were successfully performed in a patient with vEDS.
